# Perceptual pitch deficits coexist with pitch production difficulties in music but not Mandarin speech

**DOI:** 10.3389/fpsyg.2013.01024

**Published:** 2014-01-16

**Authors:** Wu-xia Yang, Jie Feng, Wan-ting Huang, Cheng-xiang Zhang, Yun Nan

**Affiliations:** ^1^State Key Laboratory of Cognitive Neuroscience and Learning, Beijing Normal UniversityBeijing, China; ^2^International Data Group/McGovern Institute for Brain Research, Beijing Normal UniversityBeijing, China; ^3^Center for Collaboration and Innovation in Brain and Learning Sciences, Beijing Normal UniversityBeijing, China

**Keywords:** congenital amusia, tone agnosia, lexical tone, musical pitch, perception, production

## Abstract

Congenital amusia is a musical disorder that mainly affects pitch perception. Among Mandarin speakers, some amusics also have difficulties in processing lexical tones (tone agnosics). To examine to what extent these perceptual deficits may be related to pitch production impairments in music and Mandarin speech, eight amusics, eight tone agnosics, and 12 age- and IQ-matched normal native Mandarin speakers were asked to imitate music note sequences and Mandarin words of comparable lengths. The results indicated that both the amusics and tone agnosics underperformed the controls on musical pitch production. However, tone agnosics performed no worse than the amusics, suggesting that lexical tone perception deficits may not aggravate musical pitch production difficulties. Moreover, these three groups were all able to imitate lexical tones with perfect intelligibility. Taken together, the current study shows that perceptual musical pitch and lexical tone deficits might coexist with musical pitch production difficulties. But at the same time these perceptual pitch deficits might not affect lexical tone production or the intelligibility of the speech words that were produced. The perception-production relationship for pitch among individuals with perceptual pitch deficits may be, therefore, domain-dependent.

## INTRODUCTION

Successful communication relies on the seamless integration of auditory perception and vocal production. It is widely acknowledged that auditory perception strongly affects vocal production. To a certain extent, impaired auditory perception hinders vocal production (Peng et al., 2004; Han et al., 2007; Xu et al., 2011). This is true for both music and language. As the two most important communication vehicles, music and language share a vital element, namely pitch. Exploring the impact of impaired pitch perception upon pitch production across music and language domains is thus the key to understanding the influence of impaired auditory perception on vocal production.

In the last decade, a developmental perceptual pitch deficit known as congenital amusia (Peretz, 2001; Peretz et al., 2002) has increasingly attracted research attention. This characteristic acoustical pitch deficit (Hyde and Peretz, 2004; Pfeuty and Peretz, 2010) was initially related to deficient musical pitch perception (e.g., Foxton et al., 2004), which occurs independently of neurological trauma, mental retardation, autism, deafness, or lack of musical exposure. Subsequent research has suggested that its origin not only is related to the impaired fine-grained pitch processing (Hyde and Peretz, 2003; Foxton et al., 2004), but also involves compromised pitch working memory (Gosselin et al., 2009; Tillmann et al., 2009; Williamson and Stewart, 2010; Williamson et al., 2010; Albouy et al., 2013), timbre perception deficits (Marin et al., 2012), and emotional prosody perception difficulties (Thompson et al., 2012).

Recent studies have evidenced amusia’s related pitch deficits in the speech domain, where pitch also plays an important role. Individuals with amusia (hereafter, “amusics”) may demonstrate lexical tone deficits among speakers of tonal (Nan et al., 2010; Liu et al., 2012) and non-tonal languages (Tillmann et al., 2011). Likewise, amusics have been shown to suffer from parallel speech intonation problems, a finding that has held true among speakers of both tonal (Jiang et al., 2010) and non-tonal languages (Liu et al., 2010). One of our earlier studies showed that a minority subgroup of amusic Mandarin speakers also had difficulty with lexical tone discrimination and identification in Mandarin speech (hereafter “tone agnosics”) (Nan et al., 2010).

This cross-domain perceptual pitch deficit offers an ideal opportunity to understand the influence of auditory perception on vocal production in music and speech. The current study sets out to investigate to what extent these perceptual pitch deficits may be related to pitch production impairments in music and Mandarin speech among Mandarin speakers. Mandarin Chinese is a tone language which relies on pitch variations to alter the meaning of words. Although amusic individuals who speak non-tonal languages also demonstrate similar problems with lexical tones (Tillmann et al., 2011), to study amusics and tone agnosics among tone language speakers will present unique perspectives on the impact of impaired pitch perception on pitch production. This is because in a tone language environment, the perception and production of lexical tones are basic communication needs of daily necessity.

In research on speakers of non-tonal languages, perceptual pitch deficits have generally been associated with poor pitch production in music (Dalla Bella et al., 2009; Hutchins et al., 2010). According to the vocal sensorimotor loop model (VSL model, Berkowska and Dalla Bella, 2009), perception is a necessary but insufficient element of vocal production. Motor components, such as motor planning and auditory-motor mapping, also play vital roles (Berkowska and Dalla Bella, 2009). In accordance with the assumptions of the VSL model, the existence of normal pitch perception may not necessarily preclude poor pitch singing, due to the possibility of independently deficient motor-related functions (Dalla Bella et al., 2007). The inclusion of both overt and covert perceptual components in the VSL model offers greater explanatory power. Within the amusic population, cases have been noted in which pitch perception impairments do not necessarily cause vocal production deficits (Loui et al., 2008; Dalla Bella et al., 2009). This could be explained by preserved covert but impaired overt pitch perceptual abilities; such an instance would corroborate the findings of previous mismatch negativity (MMN) studies on near-normal neural processing of fine-grained pitch differences without awareness in congenital amusia (Moreau et al., 2009; Peretz et al., 2009). More specifically, the auditory cortex may function relatively normally in these amusics, but its connectivity with the pars orbitalis of the right inferior frontal gyrus may function aberrantly (Hyde et al., 2011). For the other amusics, however, it is very likely that their abnormalities reside not only in the fronto-temporal pathway but also in the auditory cortices, as shown by a more recent study using magnetoencephalography and voxel-based morphometry (Albouy et al., 2013).

With regard to speech, however, linguistic tone deficits are not necessarily always correlated with production impairments, as suggested by a recent study showing that amusic speakers of non-tonal languages are unable to discriminate between speech intonations despite production being intact (Hutchins and Peretz, 2012). A more recent study, in contrast, reported impaired speech and song imitation among Mandarin speaking amusics (Liu et al., 2013). This sometimes asymmetrical perception-production relationship between music and speech domains could not be explained by the apparent acoustical difference between musical pitch and linguistic intonation – i.e., fine-grained for musical pitch but coarse-grained for linguistic intonation. As shown in one recent study (Dalla Bella et al., 2011), after controlling for acoustic pitch differences across domains, a young university student with intact musical pitch perception but impaired musical pitch imitation was shown to have intact linguistic tone production.

Among tonal language speakers, similar perceptual lexical tone deficits have been observed among individuals whose lexical tone production is intact (Nan et al., 2010). However, pitch production in music (singing abilities) among Mandarin speakers, especially among amusic and tone agnosic Mandarin speakers, has not yet been fully examined (but see Liu et al., 2013 for impaired pitch production in both music and speech for Mandarin speaking amusics only). It should be noted that, so far, the exact nature of tone agnosia is not yet clear. As shown in our early work (Nan et al., 2010), the tone agnosics had little problem identifying lexical tones carried by the same segments (e.g., word onsets and rhymes), but they had difficulties in tones embedded in different segments. This might be due to low executive or attentional control in these individuals. In the current study, we controlled these factors by matching the control, the amusic, and the tone agnosic groups on measures of executive functions and working memory. The tone agnostics were thus also amusics but with additional perceptual lexical tone disorders, as the tone agnosics and the amusics demonstrated similar levels of music perception deficits [as indicated by the similar melodic Montreal Battery of Evaluation of Amusia (MBEA) tests scores between these two groups].

In the current study, we tested musical pitch and lexical tone production among age- and IQ-matched amusics and tone agnosics (i.e., amusics who were, at the same time, tone agnosics) relative to normal controls, with three specific research aims. The first was to understand how musical pitch perception deficit is related to musical pitch production among Mandarin speakers. Second, we wanted to explore how the lexical tone impairments observed in tone agnosics would be related to musical pitch production relative to the other amusics who had intact lexical tone perception. Based on previous results of the dissociation between musical perceptual and productive abilities in amusia, we speculated that musical pitch production difficulties might be present in some but not all amusic participants. It is possible that the same holds true in tone agnosics, since they were also amusics. Alternatively, tone agnostics might show more severe musical pitch production deficits compared to the amusics, if the lexical tone deficit were detrimental to musical pitch production. The third research aim was to test lexical tone production using a novel set of objective analyses. It is possible that the subjective rating system used in our earlier study (Nan et al., 2010) has been insufficient for detecting subtle lexical tone production deficits, especially among tone agnosics.

## MATERIALS AND METHODS

### PARTICIPANTS

Sixteen amusics (six females) and twelve matched controls (five females) participated in the study. Among these 16 amusics, eight were also impaired in lexical tone perception and were thus identified as tone agnosics. A summary of all participants’ characteristics is provided in **Table 1**. All participants were university students in Beijing and native Mandarin speakers without formal musical training. They reported no vocal, neurological, or audiological deficits. Their binaural audiometric thresholds were at or below 20 dB hearing level for octaves from 250 to 8000 Hz. Additionally, the controls reported no difficulty singing. Among all participants, 23 were right-handed and five were left-handed, as assessed by the Edinburgh Handedness Inventory (Oldfield, 1971). Informed written consent was obtained from all participants. This research was approved by the Institutional Review Board of Beijing Normal University.

**Table 1:** The characteristics of the controls, the amusics, and the tone agnosics with percentages of correct responses on the MBEA and lexical tone perception tests.

	Control (*n* = 12	Amusia (*n* = 8)	Agnosia (*n* = 8)
Mean age (range)	22.5 (19–26)	21.8 (20–25)	24.5 (19–28)
Male/female	7/5	5/3	5/3
Right/left handedness	10/2	6/2	5/3
Performance IQ (SD)	115.8 (8.1)	111.3 (6.5)	110.3 (6.0)
Verbal IQ (SD)	128.8 (6.0)	126.9 (5.5)	124.9 (8.0)
Executive function (SD)	13.6 (0.9)	13.3 (0.6)	12.8 (1.1)
Working memory (SD)	15.1 (1.9)	14.1 (1.2)	13.6 (2.2)
MBEA mean (SD)			
Scale	91.7 (6.9)	60.4 (13.3)	57.4 (8.6)
Contour	90.0 (8.4)	63.3 (6.7)	55.8 (11.1)
Interval	85.8 (11.1)	60.8 (8.5)	60.4 (7.9)
Rhythm	92.8 (6.2)	65.0 (13.1)	64.3 (11.9)
Meter	81.4 (16.6)	60.4 (21.3)	65.5 (10.4)
Memory	93.4 (4.0)	74.6 (9.6)	67.6 (7.6)
Global	89.2 (6.2)	64.1 (3.0)	61.8 (5.4)
Lexical tone mean (SD)			
Mean	96.5 (4.1)	96.1 (2.8)	62.8 (12.6
Discrimination (different segments)	94.3 (4.7)	92.6 (7.3)	65.0 (8.6)

All participants were assessed with the six tests of the MBEA (Peretz et al., 2003) and the lexical tone perception tests employed in our previous study (Nan et al., 2010). The MBEA includes three melodic pitch-based tests (scale, contour and interval), two time-based tests (rhythm and meter), and one memory test. All amusic participants scored below the cut-off score of 71.7%, which corresponds to two SDs below the mean of the normal controls that was obtained in our earlier study (Nan et al., 2010). The lexical tone perception test contains identification and discrimination tasks. Among the 16 amusics, eight tone agnosics were identified based on the same criteria (i.e., performance below the cut-off scores of 79.2% for the lexical tone discrimination test with different segments – viz., word onsets and rhymes – and 80% for the average lexical tone perception tests, both of which correspond to three SDs below the means of the normal controls) as employed in our previous study (Nan et al., 2010). Except with respect to the lexical tone tests, the tone agnosics performed equivalently to the amusics on the MBEA tests (all *p*s > 0.1).

The amusic and tone agnosic groups were matched for age, handedness, performance IQ, and verbal IQ based on Wechsler Adult Intelligence Scale-Revised by China (WAIS-RC; Gong, 1992) with the control group (all *p*s > 0.1). Additionally, these two groups were also matched on measures of executive functions and working memory as derived from WAIS-RC with the control group (both *p*s > 0.1). Specifically, executive/attentional functions were indexed by the block design and similarities tests. The block design test taps attentional aspects of executive function (Chase et al., 1984; Kazui et al., 2011), whereas the similarities test may reflect abstraction and reasoning (Stern and Prohaska, 1996). The working memory index included arithmetic and digit span tests (Wechsler, 1981).

### MATERIALS AND PROCEDURES

#### Music production test

**FIGURE 1:**
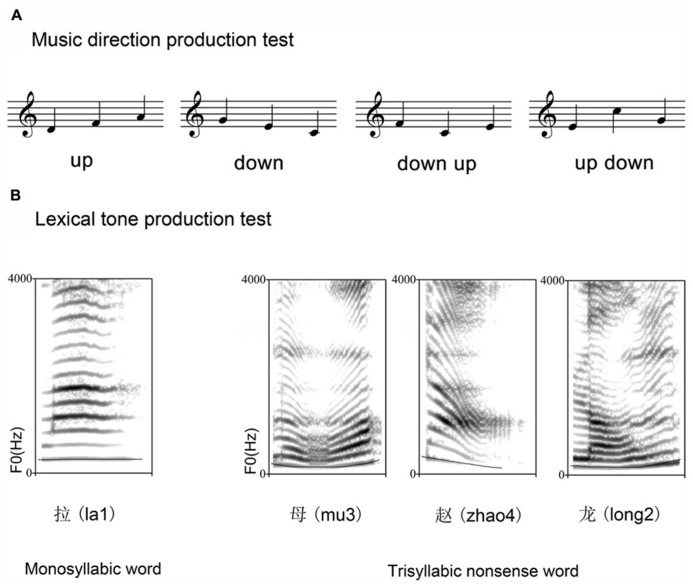
**The exemplar stimuli used in the music three-note imitation test (A) and lexical tone production test (B)**.

All of the musical stimuli were computer-synthesized with a piano-like timbre. There were two imitation conditions: one-note and three-note. One-note condition included 13 trials, each being one of the 13 notes (G3, A3, B3, C4, D4, E4, F4, G4, A4, B4, C5, D5, and E5). Each note lasted 500 ms. Three-note condition consisted of eight trials, with two trials for each of the four different directions (“up”, “down”, “down up”, and “up down”) (**Figure 1**). Except for G3, A3, and D5, the remaining notes in one-note condition were also used in three-note condition. A trial in three-note condition lasted 2500 ms, including three 500 ms notes and two 500 ms gaps in between.

#### Lexical tone production test

A female voice actor who was a native Mandarin speaker produced a list of one-syllable words for the lexical tone production tests. Recordings were made in a sound-proof booth using a Sony 60EC digital recorder and an NT1 microphone with a Samson MDR8 mixer, at a sampling rate of 44.1 kHz. There were two conditions: monosyllabic word production and trisyllabic nonsense word production. The monosyllabic word production test contained eight one-syllable words, with two lexical tones from each category (four categories: 1 = level, 2 = mid-rising, 3 = dipping, and 4 = high-falling). The trisyllabic nonsense word production test had eight trials, each formed by three one-syllable words. As a result, the trisyllabic word condition contained 24 one-syllable words in total, with six lexical tones from each tone category. For both lexical tone production conditions, the durations of the syllables (on which the lexical tones were carried) were not significantly different (mean ± SD for the monosyllabic word condition: 537.5 ± 126.9 ms; for the trisyllabic nonsense word condition: 531.7 ± 131.6 ms; *p* > 0.1 between conditions). As a result, a trial in the trisyllabic nonsense word condition lasted 2595.0 ± 225.6 ms (including two 500 ms gaps), about five times the mean duration of the monosyllabic word condition. The acoustic characteristics of the lexical tones for production tests are listed in **Table 2**.

**Table 2:** Acoustic characteristics of the lexical tones for production tests.

	Mean F0 (Hz)	F0 range (Hz)	Pitch glide size (Hz)	Duration (ms)
Tone 1	288.5 (8.1)	281.6~306.2	24.6 (11.3)	541.3 (91.3)
Tone 2	213.0 (23.1)	177.9~289.2	111.1 (20.3)	580.0 (78.0)
Tone 3	156.6 (9.0)	95.9~200.9	105.0 (23.0)	647.5 (45.0)
Tone 4	234.9 (9.9)	112.5~332.3	219.8 (31.2)	363.8 (79.5)

*The numbers in parentheses are standard deviations.*

#### Procedure

All of the words and musical stimuli were equalized for sound intensity with 10 ms linear onset/offset ramps using Praat (Boersma, 2001). All of the production tests were completed in a sound-treated booth in a single session. The stimuli were presented to the participants binaurally through Sennheiser HD 201 headphones with individually adjustable volume. Participants were asked to vocally imitate the musical note sequences or the Mandarin words that they heard as closely as possible. They were to match not only pitch (for Mandarin words the accurate lexical tonal contours were emphasized) but also tempo of the target stimuli. The order of the music production test and the lexical tone production test was counterbalanced across participants. Before each test, detailed instructions and a warm-up phase were given to ensure that all participants understood the task. In the music production test, the participants were encouraged to use syllable /la/ when imitating the note sequences. All imitation samples were recorded onto a Marantz PMD-620 digital recorder (Marantz Professional, Itasca, IL, USA), with a sampling rate of 44.1 kHz. The whole test lasted approximately 30 min for each participant.

### DATA ANALYSIS

#### Music production test

The fundamental frequency (F0) and duration of each produced note were extracted using Praat (Boersma, 2001) based on the identified steady-state phase of each sung note. Accordingly, three F0-based measures and one duration-related measure were calculated: note deviation, interval deviation, direction accuracy, and duration deviation. F0 measurements in hertz were converted to cents (100 cents = 1 semitone).

***Note deviation.*** Note deviation referred to the absolute difference between the produced F0 and the target F0 (e.g., Pfordresher et al., 2010). Octave errors were corrected.

***Interval deviation***. Interval deviation was calculated as the absolute difference between the produced interval and the target interval for the three-note condition.

***Direction accuracy***. Direction accuracy represented the rates of correctly produced directions in the three-note condition. A response was defined as correct if the successively produced three-note sequence shared the same direction as the target sequence.

***Duration deviation***. Duration deviation indicated the average absolute differences of duration between the produced note and the target note (Dalla Bella et al., 2009).

Note deviation and duration deviation were applicable for both one-note and three-note imitation conditions, whereas interval deviation and direction accuracy were entirely based on the three-note condition.

#### Lexical tone production test

***Subjective assessment***. Three independent raters (two female, native Mandarin speakers with a mean age of 24 years) classified each of the produced lexical tones from each participant as tone 1, 2, 3, or 4. When correct, the lexical tone that was produced was considered a hit. For each participant, the average scores for the monosyllabic word and the trisyllabic nonsense word production conditions were calculated separately.

***Objective analysis***. For each produced lexical tone, the mean F0, pitch glide size (i.e., the mean difference between minimum and maximum F0; Nan et al., 2010), and the duration were extracted using Praat. Octave errors were corrected for the mean F0 when necessary. These measures were then compared to those of the target lexical tones to calculate the mean F0 deviation, pitch glide size deviation, and duration deviation. All these deviation measures were calculated as the absolute difference between the produced lexical tone and the target one.

#### Statistical analysis

For all ANOVAs, the assumptions of normality and homogeneity of variance were met. If violated, then the non-parametric alternative to the planned ANOVA would be conducted instead. For all the repeated measures ANOVAs, however, an additional assumption of sphericity of the covariance matrix was also ensured. The Greenhouse–Geisser correction was applied when the sphericity assumption was violated. Bonferroni corrections were applied in multiple *post hoc* tests.

## RESULTS

### MUSIC PRODUCTION RESULTS

#### Note deviation

A mixed-model two-way repeated-measure ANOVA of note deviation with condition (2) as a within-subjects factor and group (3) as a between-subjects factor found a main effect of group [*F*(2,25) = 12.093, *p* < 0.001] and an interaction between condition and group [*F*(2,25) = 5.607, *p* = 0.01]. As shown in **Table 3**, the controls significantly outperformed the amusics and tone agnosics (both *p*s < 0.01), whereas the latter two groups performed similarly on note deviation (*p* > 0.5). Simple effect analysis of the observed interaction between condition and group suggested that the controls performed significantly better in three-note imitation condition than in one-note imitation condition (*p* < 0.01), whereas the amusics and tone agnosics both performed similarly in these two conditions (both *p*s > 0.05).

**Table 3:** The music production performances among the three groups.

	Control	Amusia	Agnosia
Note deviation (semitone)	1.9 (0.7)	2.9 (0.5)	2.8 (0.4)
Interval deviation (semitone)	1.4 (0.7)	1. 5 (0.3)	1.6 (0.8)
Direction accuracy (%)	98.9 (3.6)	75.0 (24.1)	70.3 (24.0)
Duration deviation (ms)	323.2 (171.6)	253. 5 (96.0)	229.1 (120.3)

*The numbers in parentheses are standard deviations.*

#### Interval deviation

A one-way ANOVA of interval deviation revealed no main effect of group. All three groups performed indistinguishably on interval deviation [*F*(2,25) = 1.102, *p* = 0.348; **Table 3**].

#### Direction accuracy

Direction accuracy of the controls violated the normality assumption for ANOVA (one-sample Kolmogorov–Smirnov test: *Z* = 1.837, *p* = 0.002). The Kruskal–Wallis *H* test revealed a statistically significant difference between the three groups [*H*(2) = 10.772, *p* = 0.005], with a mean rank of 19.75 for the controls, 11.44 for the amusics, and 9.69 for the tone agnosics (**Table 3**). Pairwise comparison using Mann–Whitney *U* tests suggested that the controls significantly outperformed the amusics and tone agnosics (both *p*s < 0.01), whereas the latter two groups performed similarly on direction accuracy (*p* > 0.5).

#### Duration deviation

A mixed-model two-way ANOVA of duration deviation with condition (2) as a within-subjects factor and group (3) as a between-subjects factor did not reveal any significant main effects or interactions. The three groups performed equivalently on duration deviation (**Table 3**), suggesting that, although some of the amusics and tone agnosics showed impairments on the frequency dimension of musical pitch production, their performances on the time dimension seemed relatively unaffected.

### LEXICAL TONE PRODUCTION RESULTS

#### Subjective assessment

Lexical tone production was highly accurate in all three groups, with 100% correct for both the controls and the amusics in both the monosyllabic and trisyllabic conditions. The tone agnosic group also yielded perfect lexical tone production scores for both the monosyllabic (99.0 ± 2.3%) and the trisyllabic (99.5 ± 1.4%) conditions. No main effects or interactions were observed in a mixed-model two-way ANOVA.

#### Objective analysis

Separate mixed-model three-way ANOVAs (2 conditions × 3 groups × 4 tone categories) of the results of the objective acoustic analysis, including the mean F0 deviation, pitch glide size deviation, and duration deviation, did not show any significant main effects or interactions involving group (see **Table 4**). This is consistent with the results obtained in the subjective assessments, suggesting that neither the tone agnosics nor the amusics were impaired in lexical tone production relative to the controls.

**Table 4:** The results of the objective analysis for the two lexical tone production tests across the three groups.

	Control	Amusia	Agnosia
**Mean F0 deviation (semitone)**
Tone 1	1.9 (1.7)	2.9 (1.7)	1.9 (1.4)
Tone 2	2.5 (1.4)	3.3 (1.1)	2.2 (1.1)
Tone 3	3.4 (0.9)	3.7 (1.4)	4.0 (0.8)
Tone 4	2.6 (1.6)	2.7 (1.4)	2.1 (1.5)
**Pitch glide size deviation (semitone)**
Tone 1	0.7 (0.3)	0.6 (0.3)	0.9 (0.5)
Tone 2	2.4 (0.9)	2.4 (1.3)	2.4 (0.8)
Tone 3	5.7 (1.4)	6.7 (1.6)	5.4 (2.0)
Tone 4	7.9 (1.7)	9.3 (2.1)	7.5 (2.3)
**Duration deviation (ms)**
Tone 1	92.8 (45.6)	78.8 (42.1)	94.0 (32.7)
Tone 2	106.2 (42.4)	96.2 (30.5)	57.5 (39.5)
Tone 3	131.7 (66.4)	113.2 (84.6)	91.6 (50.0)
Tone 4	107.8 (68.6)	106.6 (57.7)	83.1 (35.5)

*The numbers in parentheses are standard deviations. Tone 1 is the level tone, tone 2 mid-rising, tone 3 dipping, and tone 4 high-falling.*

### CORRELATION ANALYSIS

Spearman’s Rank Correlation analyses were conducted to examine the relationship between musical pitch perception, lexical tone perception, musical pitch production, and objective lexical tone production measures across and within the three groups of participants. The subjective lexical tone production scores were not taken into account due to the ceiling effect. Pitch perception measures included the melodic MBEA scores (averaged across the scale, contour, and interval tests) and the average lexical tone perception scores. Pitch production measures included note deviation, interval deviation, direction accuracy, and duration deviation for music as well as the mean F0 deviation, pitch glide size deviation, and duration deviation for lexical tones.

**FIGURE 2:**
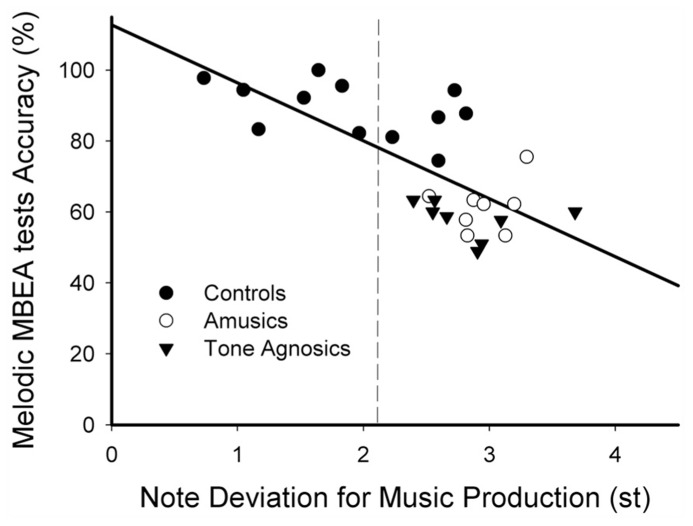
**A significant negative correlation between the melodic MBEA score and note deviation for music production tests among the three groups.** A dashed line divides two distinct groups based on individual performances on note deviation for the controls, the amusic, and tone agnosic groups.

The results showed that the melodic MBEA scores were positively correlated with the average lexical tone perception scores across the three groups [*r*_s_(28) = 0.575, *p* = 0.001]. More importantly, the melodic MBEA scores were also significantly and negatively correlated with note deviation across the three groups (**Figure 2**), *r*_s_(28) = -0.695, *p* < 0.001. However, neither of these two correlations held within each individual group (all *p*s > 0.1). There was no significant correlation between pitch perception measures (both melodic MBEA scores and the average lexical tone tests scores) and other music pitch production measures (including direction accuracy, interval deviation, and duration deviation) or lexical tone production measures (all *p*s > 0.1). There was no significant correlation between the equivalent music production and perception measures (i.e., direction accuracy and MBEA contour score or interval deviation and MBEA interval score) either (both *p*s > 0.1).

These results suggest that musical pitch perception is tightly linked to lexical tone perception, and musical pitch production and perception are significantly correlated (**Figure 2**).

### INDIVIDUAL-LEVEL ANALYSIS

We conducted individual-level analyses based on note deviation and direction accuracy; we selected these two measures on account of their clear group differences.

For all 28 participants from the three groups, K-means cluster analysis using note deviation yielded two distinct groups (good pitch imitation vs. poor pitch imitation). As shown in **Figure 2**, a subgroup of the controls (*n* = 7) imitated musical pitch with significantly smaller note deviations (around 1.4 semitones) than the rest of the controls and all the amusics and tone agnosics (around 2.8 semitones).

**FIGURE 3:**
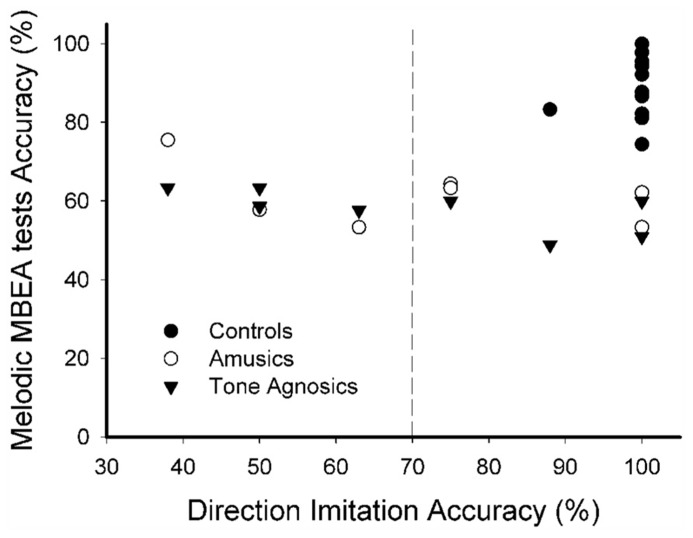
**Direction accuracy of three-note imitation across the three groups.** A dashed line divides two distinct groups based on individual performances on direction accuracy of three-note imitation for the controls, the amusic, and tone agnosic groups.

On the other hand, K-means cluster analysis using direction accuracy yielded two different distinct groups. As shown in **Figure 3**, all controls (*n* = 12), five amusics, and four tone agnosics demonstrated relatively better music direction production performance (i.e., the “good direction imitation” group, with direction accuracy around 95%), whereas three amusics and four tone agnosics fell into the other group (the “poor direction imitation” group, with direction accuracy around 50%).

These results suggest that note deviation and direction accuracy are two different measures describing musical pitch production. Measured by note deviation, five controls and all the amusics and tone agnosics demonstrated poor musical production, whereas measured by direction accuracy, none of the controls and only subgroups (about half) of the amusics and tone agnosics showed poor musical production.

## DISCUSSION

Auditory perception and vocal production are closely related, although the exact nature of this link is still hotly debated (e.g., Lotto et al., 2009; Dalla Bella et al., 2011). The present study tried to determine to what extent perceptual pitch deficits across domains are related to pitch production difficulties in music and Mandarin speech. Our results showed that the amusics and tone agnosics both had musical pitch production difficulties. However, the perceptual pitch deficits across domains did not affect lexical tone production.

The current study found that the controls outperformed both the amusic and tone agnosic individuals on note deviation and music direction accuracy. Moreover, across the three groups, note deviation performance was significantly and negatively correlated with the melodic MBEA scores, suggesting an association between musical pitch perception and production. These findings corroborates previous results found among speakers of non-tonal languages (Dalla Bella et al., 2009) and further supports the notion that pitch perception and pitch production are closely coupled in the music domain (Berkowska and Dalla Bella, 2009; Dalla Bella et al., 2011) by using data from speakers of a tonal language. Furthermore, there was also a close link between lexical tone and musical pitch perception, corroborating the notion that lexical tone deficits among tone agnosics are associated with musical pitch disorders (Nan et al., 2010). However, it should be noted that according to the current results, tone agnosics were not more impaired than the amusics in musical pitch production, suggesting that lexical tone perception deficits are not necessarily detrimental to musical pitch production.

More interestingly, all participants with lexical tone perception impairments demonstrated intact lexical tone production, corroborating our previous results (Nan et al., 2010). This dissociation between perception and production of linguistic tones among amusics is also in line with previous results obtained using speakers of non-tonal languages (Hutchins and Peretz, 2012). A recent event-related potential paradigm (ERP) study reports a similar dissociation between production and perception of lexical tones for Cantonese (Law et al., 2013), suggesting that the observed independence between perception and production for lexical tones is relatively robust. This is partly in line with results from a recent study, which showed partial support for domain specific pitch processing, but at the same time a close association between song and speech imitation performance (Mantell and Pfordresher, 2013).

Nonetheless, neither the VSL model (Berkowska and Dalla Bella, 2009) nor the vocal-motor encoding theory (Hutchins and Peretz, 2012) can easily incorporate the distinct perception-production relationships for music and Mandarin speech that were observed in the current study. It is possible that pitch production in music and Mandarin speech involve independent but interactive systems, similar to the recently proposed dual routes for verbal repetition (Yoo et al., 2012). The production of lexical tones may mainly engage the acoustic–phonetic systems primarily relying on the high temporal resolution of the left hemisphere, whereas the production of musical pitches as well as cross-domain pitch perception (including both musical pitch and lexical tone perception) may mainly require the high frequency resolution of the right hemisphere (Zatorre and Belin, 2001; Luo et al., 2006), although the left inferior frontal gyrus is often implicated in lexical tone (Hsieh et al., 2001) or lexical tone and music pitch perception (Nan and Friederici, 2013) in Mandarin speakers as well. Thus, this classical view of hemispheric asymmetries in spectral and temporal processing (Zatorre and Belin, 2001) may account for how Mandarin lexical tone production can be preserved simultaneously with impaired production in musical pitch and impaired pitch perception across domains.

Moreover, the current results provide more insights on the nature of congenital amusia from the perspective of music production. Both the amusics and tone agnosics performed similarly to the controls on three-note imitation when measured by interval deviation, but these two groups were significantly impaired relative to the controls as measured by direction accuracy. This corroborates the accumulating results on music perception: compared to the controls, the amusics are not necessarily impaired in pitch discrimination thresholds (Foxton et al., 2004; Tillmann et al., 2009; Albouy et al., 2013), but they show relatively consistent difficulties in discriminating pitch direction (Foxton et al., 2004; Liu et al., 2010). Hence, the current study adds more evidence on the notion that the related deficits of congenital amusia may as well arise at a relatively higher stage of pitch processing, e.g., perceiving and producing pitch directions (for a review, see Stewart, 2011). Furthermore, the observed clear split of good and poor musical pitch production groups among amusics and tone agnosics as measured by music direction accuracy is in line with previous results on the possible existence of subgroups within the amusic population (Dalla Bella et al., 2009; Nan et al., 2010), converging on the notion that congenital amusia is indeed a complex disorder and often involves variously mixed presentations (Stewart, 2011).

It should be noted that, in the current study, all of the participants’ performances in musical pitch production (except direction accuracy) were relatively lower when compared to previous similar studies (e.g., Amir et al., 2003; Watts et al., 2005; Dalla Bella et al., 2007; Pfordresher et al., 2007; Wise and Sloboda, 2008). This may be due to the effects of tasks and stimuli for the music production tests. First, despite using similar stimuli, our imitation task was more demanding compared to the tasks used in previous studies (Amir et al., 2003; Watts et al., 2005; Pfordresher et al., 2007). These previous studies usually presented each stimulus several times during the test, whereas in the present study, all stimuli were presented only once. Second and more importantly, for music production tasks, the present study used piano tones in a pitch range that was suitable for females but not for male participants. As we have more male participants (*n* = 17) than female participants (*n* = 11), this gender effect would have contributed to the overall lower musical production performance observed in the current study. However, it should be noted that, when gender was considered as an additional between-subjects factor, no significant main effect or interactions involving gender were found, probably due to the small sample size. Likewise, in lexical tone production task, female voices were used. This might also have caused male participants more difficulties in lexical tone imitation than females, although no gender effect was statistically significant.

Additionally, it is important to point out the fact that the lower production performances we observed occurred not only for the pitch dimension but also for the time dimension, in contrast to the findings of previous research (Dalla Bella et al., 2009). Based upon the same criterion, the current study found an average duration accuracy of approximately 30% across the three groups, whereas a previous study (Dalla Bella et al., 2009) with a familiar melody reported average duration accuracy more than 90% among the controls and the amusics. Nonetheless, the current data demonstrated that the amusics as well as tone agnosics were not impaired relative to the controls in the time dimension for music production, corroborating the previous study (Dalla Bella et al., 2009). Together with data from musical pitch perception (Hyde and Peretz, 2004; Nan et al., 2010), the current results further support the notion that pitch deficits in both perception and production related to congenital amusia mainly affect the frequency dimension but not the time dimension (Hyde and Peretz, 2004; Dalla Bella et al., 2009; Nan et al., 2010).

It should also be noted that a more recent study reported impaired speech and song imitation among Mandarin-speaking amusics (Liu et al., 2013), whereas our results showed that the amusics were only impaired in musical pitch production but not lexical tone production. This discrepancy of speech tone production in Mandarin-speaking amusics between our results and those of Liu et al. (2013) might be caused by different stimuli employed in these studies. The current study used speech stimuli which contained equal numbers of four lexical tones in Mandarin, whereas Liu et al. (2013) did not control for the number of lexical tones from each tone category. As shown in **Table 2** (Liu et al., 2013), among the set of selected speech stimuli used in the experiment, there were 28 level tones (tone 1) and five dipping tones (tone 3). With such a high rate of level tones (28 among 60 syllables, almost half), the speech stimuli did not well represent Mandarin which has four main lexical tones.

The observed intact lexical tone production among amusics and tone agnosics in our present study, however, might also be due to the fact that the current speech stimuli were mainly drawn from everyday materials. It is inevitable that the speech stimuli employed in the current study were more familiar to the participants than the music stimuli. Over years of experience with daily production needs, amusics and especially tone agnosics might have learned how to produce speech tones despite their perceptual impairments. But clearly the extent of their exposure and daily production pressure for music pitches is much lower. The resulted disparity of the learning processes between music and speech domains might thus also account for the intact lexical tone production but impaired music pitch production among amusics and tone agnosics relative to controls. Additionally, the timbre difference between the speech (human voices) and music stimuli (piano tones) might have played a role. As suggested by previous research (e.g., Leveque et al., 2012), piano timbre is more difficult to imitate than human voice.

Nonetheless, it should be noted that although the F0 deviations of the produced lexical tones in all the three groups were not negligible, the intelligibility of the speech words were not affected. This indicates different functional standards for pitch production in lexical tones than that in music. More interestingly, based on the current results, we might tentatively speculate that the pitch-related production skills necessary for intelligibility of speech (such as those measured by direction accuracy and interval deviation) are largely intact in the amusics and tone agnosics as tested in the current study, while the aspect that is more specific to music (for instance the one represented by note deviation) is the one that is most compromised.

## CONCLUSION

Individuals with perceptual pitch deficits known as congenital amusics and tone agnosics represent unique opportunities to understand the intriguing relationships between pitch production and perception in music and language. The current study examined to what extent the perceptual pitch deficits involved in both the amusics and tone agnosics are related to pitch production difficulties in music and Mandarin speech among Mandarin speakers. For music, our results demonstrated that pitch production difficulties may be present in both the amusic and tone agnostic groups, resulting in significantly enlarged note deviation and decreased direction accuracy in these two groups relative to the controls. Moreover, tone agnosics were not more impaired than the amusics in musical pitch production, suggesting that lexical tone perception deficits are not necessarily detrimental to musical pitch production. For language, on the other hand, all three groups were able to imitate lexical tones with perfect intelligibility, suggesting that the perceptual pitch deficits across domains may coexist with intact lexical tone production. Taken together, the current results imply that the perception-production relationship for pitch among individuals with perceptual pitch deficits may be domain-dependent.

## Conflict of Interest Statement

The authors declare that the research was conducted in the absence of any commercial or financial relationships that could be construed as a potential conflict of interest.
